# Tech mining: a revisit and navigation

**DOI:** 10.3389/frma.2024.1364053

**Published:** 2024-04-29

**Authors:** Alan L. Porter, Yi Zhang, Nils C. Newman

**Affiliations:** ^1^Search Technology, Inc., Peachtree Corners, GA, United States; ^2^Technology Policy and Assessment Center, Georgia Institute of Technology, Atlanta, GA, United States; ^3^Faculty of Engineering and Information Technology, Australian Artificial Intelligence Institute, University of Technology Sydney, Ultimo, NSW, Australia

**Keywords:** tech mining, technology opportunities analysis, competitive technical intelligence, science, technology and innovation, future-oriented innovation pathways

## Abstract

This mini-review arrays the pertinent tools and purposes of “Tech Mining” – shorthand for empirical analyses of Science, Technology and Innovation (ST&I) data. The intent is to introduce the range of tools, and show how they can complement each other. Tech Mining aims to generate powerful intelligence to help manage R&D and innovation processes. We offer a 5-part array to help relate the analytical elements. An overview of a case study of Hybrid and Electric Vehicles illustrates the complexities involved and the potential to generate valuable “intel.”

## Introduction

“Tech Mining” is short for the analytical combination of bibliometrics [counting research and development (R&D) activities] and text analyses of Science, Technology and Innovation (ST&I) information resources. ST&I information can be drawn from various sources, but especially from topical searches of R&D publication and patent databases (e.g., *Web of Science; Derwent World Patents Index*). Tech Mining can be done for a range of purposes – particularly including competitive technical intelligence to track possible competitors and/or collaborators, or to profile R&D for scholarly studies for publication. Tech Mining is vital to project emerging technologies. Such empirical results can inform R&D prioritization and innovation management.

Tech Mining, as initially defined in the book— *Tech Mining: Exploiting New Technologies for Competitive Advantage–* by Porter and Cunningham (2005), systematically defined an analytical framework of retrieving technological information from ST&I data sources. Since the late 2000s, with close interactions with technology opportunities analysis (Porter and Detampel, 1995), tech mining has become one of the most significant analytical approaches for profiling technological areas (Guo et al., 2010) and informing technology R&D (Porter and Newman, 2011) by understanding “WWWW”— who is doing what, where and when?— in its technology space. Comprehensive communications between quantitative-approach-based tech mining tools and expert-knowledge-based, qualitative approaches created great potential to investigate technological forecasting through concepts and systematic tools of technology management, e.g., forecasting innovation pathways (FIP) (Robinson et al., 2013), technology roadmaps (Zhang et al., 2013; Huang et al., 2014), technology delivery systems (TDS) (Guo et al., 2012; Huang et al., 2018), and technology life cycle analyses (Huang et al., 2020). Consequently, tech mining's functional accessibility with science maps, including co-occurrence maps and science overlay maps (Rafols et al., 2010) further enabled visualization and network analytics in the late 2010s (Zhang et al., 2018b; Zhou et al., 2019).

Recently, the tech mining community also expressed great passion to elaborate cutting-edge artificial intelligence (AI) and data analytical techniques, from word embedding (Zhang et al., 2018a), steaming data analytics (Zhang et al., 2017), to heterogeneous and dynamics network analytics (Huang et al., 2021; Zhang et al., 2022b), and graph representation learning (Choi et al., 2022).

While it has been nearly 20 years since the launch of the tech mining story, it is time to revisit what the community has achieved and help navigate some potential future directions. Targeting its community and broad audiences in technology management, ST&I studies, and bibliometrics/scientometrics, this paper briefly summarizes the general concepts, tasks, and tools of tech mining. It ties it to the context of ST&I management through a case example on hybrid and electric vehicles (HEVs). We discuss current interplays between AI tools and tech mining, addressing some open questions such as tech mining's elaboration with knowledge graphs and large language models.

## Tech mining: concepts, tasks, and tools

The book – *Tech Mining: Exploiting New Technologies for Competitive Advantage* – by Porter and Cunningham (2005) provides the foundation for tech mining. The book is organized in two parts. The first part (Chapters 1-5) introduces the principles of such analyses and how they help fulfill aims of translating ST&I advances to successful technology commercialization more effectively. The second part (Chapters 6-16) details how to perform Tech Mining. Topics include identifying prime data resources and retrieving the pertinent information, often as abstract record compilations. Basic analyses address the reporters' questions – *Who is doing What, Where, and When?* (Answering *How?* and *Why?* questions needs additional human insights, and might be aided by AI). Answers need to be reported effectively via multiple modes – usually incorporating visualizations. Such empirical analyses can provide valuable *research profiling*. The book lays out R&D publication and patent analyses, and guides how to track changes over time (trend analyses). It offers a framework of 13 management issues, within which one can distinguish some 39 questions for Tech Mining, pointing toward over 200 *innovation indicators*. The book also extends advice on managing the Tech Mining process and on evaluating results.

Tech Mining combines data mining tools with ST&I domain knowledge. Performing it calls for data manipulation, statistical analyses, specialized text analyses, visualizations, and report generation. One can draw on general purpose software tools to do these (e.g., *MicroSoft Excel, R, Python*). But the book leans heavily on *VantagePoint* (www.theVantagePoint.com, or closely related, *Derwent Data Analyzer*) software expressly developed for Tech Mining. These programs have routines for combining data sets, consolidating name variations (e.g., topical variants), and extracting data subsets for in-detail analyses. The software enables one to list top items in target fields (e.g., leading authors), cross two fields against each other as a matrix to seek relationships (e.g., leading inventors by patent classes), cluster entities in a field, categorize records, or generate data maps (e.g., present the network of co-cited authors to ascertain patterns of shared interests). Advanced tools include generation of emergence indicators, various specialized visualizations, and reporting tools to facilitate generation of tables and charts.

Terminology and emphases vary; “Tech Mining” is only one descriptor of analyses of ST&I information resources. Another term, of which to be cognizant of tools and findings, is “research profiling” (Porter et al., 2002). Search in Google Scholar finds ~2200 “tech mining” hits and ~2390 “research profiling” mentions (December 29, 2023). The Introduction and Acronyms list note several related labels for various ST&I analyses.

## Tech mining in the context of ST&I management

**Figure 1:**
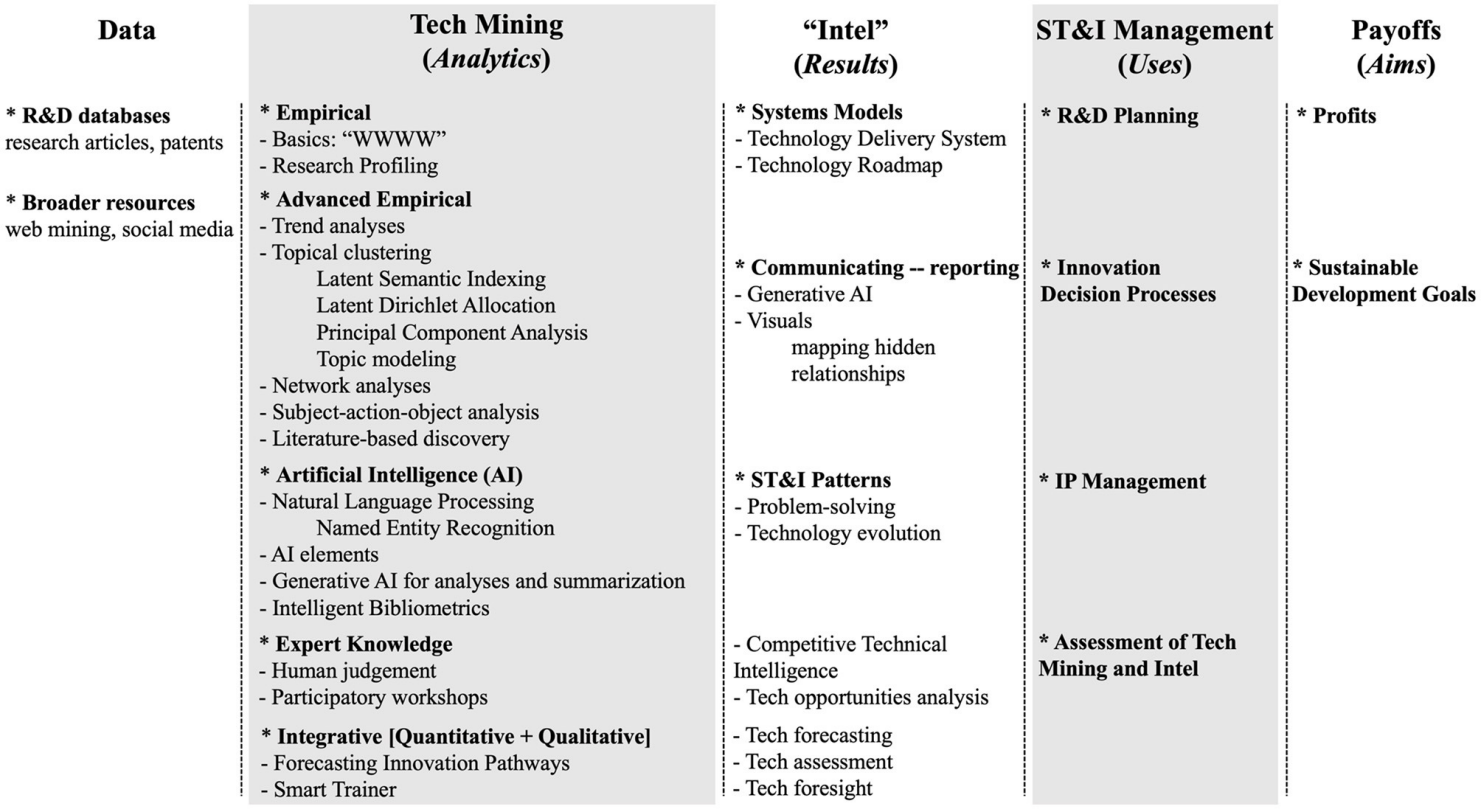
Tech mining in the context of technology management.

Figure 1 arrays elements pertinent to *analysis of emerging technologies* [see also “future-oriented technology analysis (FTA**)**” (Cagnin et al., 2008)]. Location of the elements is somewhat arbitrary – items are not aligned across columns. The Figure aims to alert the reader to the variety of ST&I analytics and uses for them. A danger lies in having rich data resources with an abundance of analytical tools that tempt us to generate too many results and pretty visualizations that lack focus on ST&I management uses.

Figure 1 is structured in five columns that attempt to categorize important related elements:

1. **Data** (information resources) – we note two main types (of many possible resources); one needs to consider how much data is enough for the task at hand.
2. **Tech Mining** (analytics) – this is the focus of this mini-review; here we note a range of tools that one could use to refine, analyze, and visualize elements extracted from the data being used. One purpose in presenting Figure 1 is to remind that the analytics are a means to various ends (suggested in the next three columns). We note “Integrative” procedures that draw together empirical, data-based findings and expert judgment, to gain the best of both. Our HEV case study on “Forecasting Innovation Pathways” will illustrate.
3. “**Intel**” (i.e., intelligence – valuable analytical results) – that serve intended uses and aims (the last two columns). The items that we list are not comprehensive, just suggestive of some considerations in applying Tech Mining. We mention systems models to encourage consideration of organizational capabilities and external socio-economic forces that bear upon a technology under study and its prospects for success (e.g., commercial innovation). We mention Generative AI to call attention to potential AI uses in associating results, and further discuss the interplays between AI tools and tech mining in the Future Directions section.
4. **ST**&**I Management** (uses) – in the last two columns we merely offer a few ideas on using Tech Mining results. Under Uses, we nominate planning (prioritizing, organizing) an organization's R&D. Innovation decision processes entail translating one's R&D results into effective research, commercial, or governmental applications. Intellectual property (IP) (especially patent) can use Tech Mining results to help gain value from those resources. Assessment of results can range from checks on their validity and robustness to exploration of intended and unintended, and direct and indirect implications.
5. **Payoffs** (aims) – by making better informed decisions, one may well aspire to increase profits in various ways. We mention Sustainable Development Goals (SDGs) to encourage consideration of sustainability with respect to one's ST&I activities.

Examining 177 Web of Science (WoS) abstract records from a search on either “Tech Mining” or “research profiling” – alerts us to some associated keywords: technical intelligence, science mapping, and Semantic TRIZ. From our experience, in recent years, we see increasing Tech Mining engagement of: *intelligent bibliometrics* (incorporating various AI capabilities) (Zhang et al., 2020, 2021a), *knowledge modeling* (to identify related research, not limited to use of particular terms in searching) (Cassidy, 2020; Wu et al., 2023), and *Literature-Based Discovery* (to identify related research falling outside one's topical search domain) (Porter et al., 2020; Zhang et al., 2023b).

To locate related research not constrained to papers using the term “Tech Mining” per se, we examine papers associated with the thirteen annual Global Tech Mining (GTM) Conferences (through 2023, and continuing). We located DOIs for 151 related papers associated with those GTM conferences. A WoS citation report on these 151 provides a perspective on the scientific communities pursuing related interests:

- Leading Web of Science Categories of the citing papers are Environmental Sciences, Information Science/Library Science, and Business/Management.
- China and the USA most fund these citing papers (National Natural Science Foundation of China and the U.S. National Science Foundation).
- The citing research is most associated with Sustainable Development Goals (SDGs) 09 – Industry Innovation and Infrastructure, and 13 – Climate Action.

And, a sampling of nine GTM papers with over 40 cites gives a sense of Tech Mining's topical spread:

1. Clustering scientific documents with topic modeling by Yau et al. (2014) (*Scientometrics*)
2. Spaces for sustainable innovation: Solar photovoltaic electricity in the UK by Smith et al. (2014) (*Technological forecasting and social change*)
3. A patent analysis method to trace technology evolutionary pathways by Zhou et al. (2014) (*Scientometrics*)
4. A hybrid visualization model for technology roadmapping: bibliometrics, qualitative methodology and empirical study by Zhang et al. (2013) (*Technological analysis and strategic management*)
5. Parallel or Intersecting Lines? Intelligent Bibliometrics for Investigating the Involvement of Data Science in Policy Analysis by Zhang et al. (2020) (*IEEE Transactions on Engineering Management*)
6. How competitive forces sustain electric vehicle development by Wesseling et al. (2014) (*Technological forecasting and social change*)
7. Four dimensional Science and Technology planning: A new approach based on bibliometrics and technology roadmapping by Huang et al. (2014) (*Technological forecasting and social change*)
8. Big Pharma, little science? A bibliometric perspective on Big Pharma's R&D decline by Rafols et al. (2014) (*Technological forecasting and social change*)
9. The state-of-the-art on Intellectual Property Analytics (IPA): A literature review on artificial intelligence, machine learning and deep learning methods for analysing intellectual property (IP) data by Aristodemou and Tietze (2018) (*World patent information*)

## Showcases of tech mining: forecasting innovation pathways

We sought a case study to illustrate how Tech Mining and associated analytics can be incorporated with human knowledge to assess an emerging technology and associated opportunities. Robinson et al. (2013) introduce the “FIP” framework. That paper presents two case analyses – deep brain interface devices, and U.S. nanobiosensors (Figure 4 there connects each of four nanostructures, via functions they serve, to develop products, that selectively support different applications).

**Figure 2:**
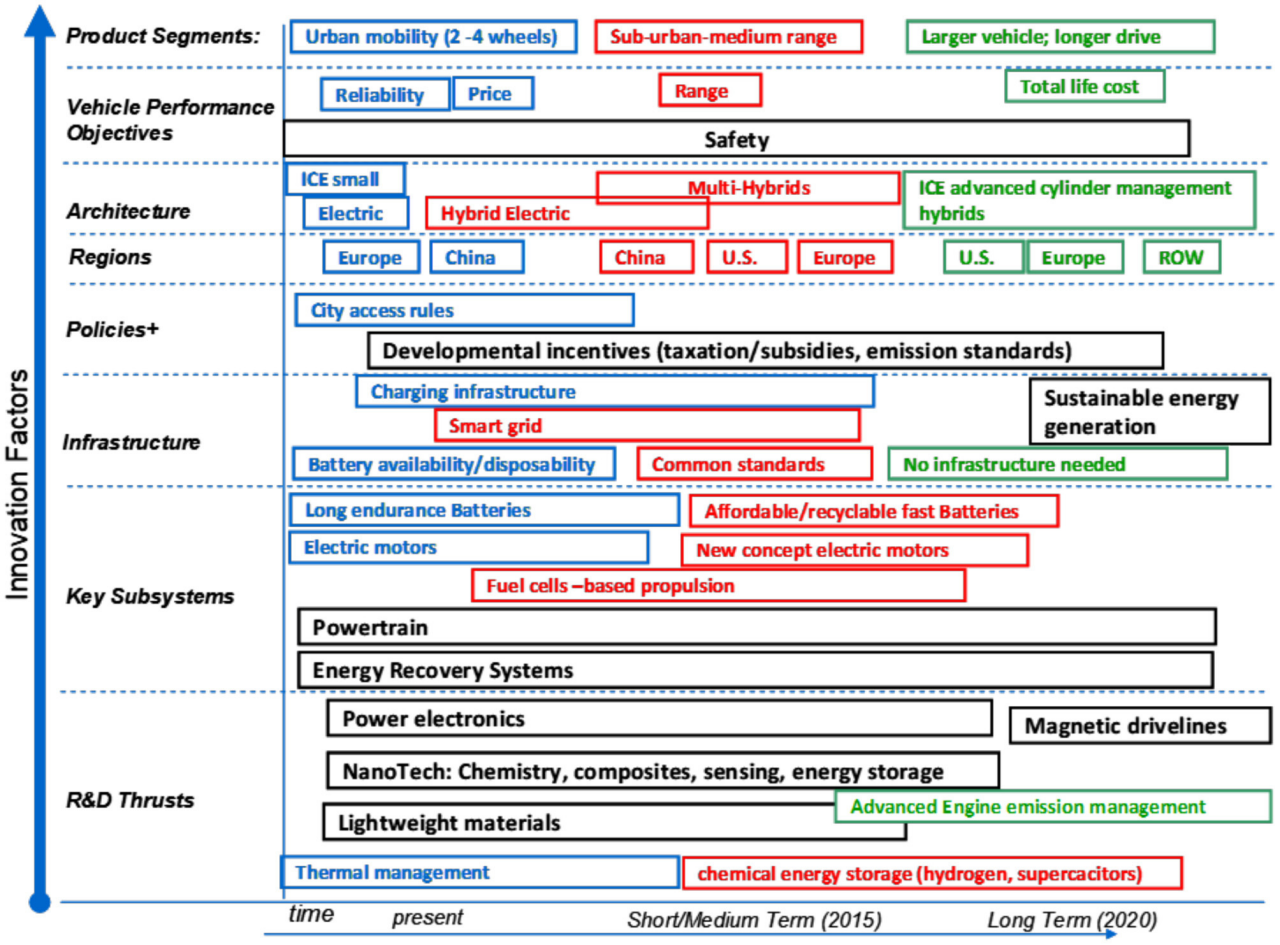
Final workshop “future innovation pathways” for HEVs (Porter et al., 2013).

In Figure 2, we highlight another FIP exercise done with the Swedish company, SKF. We draw from two versions (Porter et al., 2013, 2015). This study is interesting in combining empirical Tech Mining analyses with expert opinion gained via a focused workshop.

SKF conducted an FIP exercise on HEVs. HEVs combine multiple sub-systems, advancing at different rates technologically, with complex technical and market infrastructures. For instance, Asian automotive production and markets appear vital for the future of HEVs, and various technologies and applications (e.g., two-wheelers) warrant tracking.

The FIP project entailed four process stages that we briefly treat:

1. Understand the target technology (i.e., HEVs) and its Technology Delivery System.
2. **Tech mining** – profile relevant R&D and key players; identify potential applications.
3. Forecast innovation paths – lay out alternative paths; explore components and dependencies; perform technology assessment; engage experts.
4. Synthesize and report to different audiences.

Stage 1 – TDS – identifies key system players; it interweaves EVs and hybrid vehicles, showing complementarities (shared technological and market components), but, also, competition. Multiple HEV technological and contextual forces are rapidly evolving.

Stage 2 – Tech Mining – patent analyses led to a landscape map that helped define major subsystems. Additional emphases included answering “WWWW” (Who, What, Where, When) for HEV R&D, including:

- Prominent actors – e.g., top publishing universities in five key HEV subsystems
- Potential applications – see Figure 2, facilitated by tracking R&D emphases over time.

Stage 3 – FIP – The project organized a workshop to integrate multiple human perspectives with empirical intelligence. The workshop split into small groups to address three priority market segments and three prime geographical regions, then regrouped to review and develop consensus re: opportunities for SKF. Manifold factors influence HEV innovation paths, so technology delivery systems are complex.

Figure 2 (from the 2013 paper) shows the FIP template after extensive workshop revision of the initial version presented to them. Note, for instance, influence of product segmentation (top row) ‘driving' vehicle performance objectives, and on down through key tech subsystems, to key R&D thrusts. What is desired for urban mobility differs markedly from what long range heavy trucking needs. The requirements of future military vehicles offer another different “driver” re: HEV development and deployment.

Stage 4 – Conveying Findings – Results were consolidated for each of three target market segments and for three target regions. Regional differences (among the U.S., Europe and Asia) reflect in altered roles for key players – e.g., Original Equipment Manufacturers – and governments interact variously with technological developments (no worldwide standards are set).

Consider one segment – use of HEVs in urban areas. This is affected by trends, including growth in global urban population, climate concerns, and parking availability. Alternative modalities will compete (e.g., electric 2-wheelers); vehicle range matters (as urban regions grow); infrastructure (recharging) is critical. Workshop exploration of the pertinent TDS features noted that city governments exert strong influence (e.g., what are allowable vehicle power systems and sizes?); ownership models could change; and customer values are apt to evolve rapidly (It is interesting to revisit findings a decade since the study as EVs “take off,” rapidly passing hybrids).

## Future directions of tech mining: a vision with artificial intelligence

Tech Mining uses bibliometrics and text analytics especially to garner insights from R&D publications and patents. To do so, it can gain extraordinary benefits from the emergence of AI, with increased computational capabilities in analyzing large-scale ST&I data, discovering complicated ST&I patterns, and forecasting related activities. We summarized this impressive direction as intelligent bibliometrics in Figure 1, and collected such AI-empowered endeavors in our journal special issues, for advanced tech mining (Zhang et al., 2022a), big data-driven tech mining (Huang et al., 2022b), etc. Moreover, the “AI + Informetrics” workshop series further extends the utilization of AI-enhanced bibliometric models to broad ST&I interests (Zhang et al., 2023c). The infusion of AI techniques into Tech Mining has become vital. Embedding techniques have become essential for identifying technological topics (Zhang et al., 2018a). Network analytics bring effective tools to analyze ST&I networks (Huang et al., 2022a) and diversifies the measures of technological characteristics and emergence (Zhang et al., 2021b). Interest to utilize knowledge graphs (i.e., heterogeneous ST&I networks) to understand emerging technologies is on the rise (Lee et al., 2022; Choi et al., 2023).

Such activities inspire us to anticipate a few future pathways of Tech Mining with AI.

AI as an intelligent tool to achieve advanced Tech Mining: We have already observed such interactions in our community's current endeavors, and AI has demonstrated impressive analytical capabilities in answering the fundamental questions about Who, What, Where, and When? Despite potential challenges in understanding complicated ST&I patterns, we anticipate in-depth engagement between Tech Mining and AI in proposing novel solutions for the How and Why questions re: ST&I development. Our attempt to develop a heterogeneous knowledge graph mining approach to track knowledge trajectories is one example (Zhang et al., 2023b).

We consider AI to be a game-changer of the paradigm of Tech Mining and ST&I management. ChatGPT triggered societal awareness of the Pandora's Box of AI, and the incredible power of large language models (LLMs) is revolutionizing the thinking modules and analytical processes of human beings. The computer science community is working on the synergy of LLMs with knowledge graphs (Pan et al., 2023) and the exploitation of such capabilities for broad tasks in information extraction, summarization, and annotation (Zhang et al., 2023a).

Tech Mining invites a hybrid approach, with quantitative and qualitative methodologies. We wonder whether a new paradigm may minimize human knowledge and more heavily rely on intelligent machines and models?

A first pathway forward could be continuous, with the Tech Mining community already in a transformative process to adapt to these new capabilities. Alternatively, a second pathway would be disruptive and may re-build the entire Tech Mining ecosystem. We thus urge our community, and those analyzing technological emergence in other terms, to prepare for such a challenging, but exciting, future.

## Conclusions

This mini-review seeks to bring together various elements of analyzing emerging technologies. Figure 1 arrays five key ingredients: Data, Tech Mining, “Intel,” ST&I Management, and Payoffs. Within that framework, we position Tech Mining as the core analytics to provide key empirical results concerning a target ST&I topic under study. Tech Mining is complemented by human judgment (experiential knowledge and preferences). This mini-review also re-visits its evolution over the past two decades and its interplays with a range of theories, concepts, and tools, crossing multiple disciplines including technology management, bibliometrics, computer science, etc. Our case example on HEVs demonstrates a study of technological forecasting by utilizing Tech Mining and related analytical tools. While AI is increasingly enriching that juxtaposition of empirical and expert ST&I knowledge, we open discussion on some future pathways of Tech Mining with AI, to prepare for this game-changer.

## Author contributions

AP: Conceptualization, Writing—original draft, Writing—review & editing. YZ: Conceptualization, Methodology, Writing—review & editing. NN: Conceptualization, Project administration, Writing—review & editing.
